# A novel flavobacterial phage abundant during green tide, representing a new viral family, *Zblingviridae*

**DOI:** 10.1128/aem.00367-24

**Published:** 2024-07-02

**Authors:** Xiaoyue Guo, Xinran Zhang, Hongbing Shao, Andrew McMinn, Yantao Liang, Min Wang

**Affiliations:** 1 College of Marine Life Sciences, Institute of Evolution and Marine Biodiversity, MOE Key Laboratory of Evolution and Marine Biodiversity, Frontiers Science Center for Deep Ocean Multispheres and Earth System, Center for Ocean Carbon Neutrality, Ocean University of China, Qingdao, China; 2 UMT-OUC Joint Centre for Marine Studies, Qingdao, China; 3 Institute for Marine and Antarctic Studies, University of Tasmania, Hobart, Tasmania, Australia; 4 Haide College, Ocean University of China, Qingdao, China; 5 The Affiliated Hospital of Qingdao University, Qingdao, China; Colorado School of Mines, Golden, Colorado, USA

**Keywords:** flavobacterial phage, siphovirus, *Ulva prolifera*, genomic and phylogenetic analysis, distribution

## Abstract

Flavobacteriia are the dominant and active bacteria during algal blooms and play an important role in polysaccharide degradation. However, little is known about phages infecting *Flavobacteriia*, especially during green tide. In this study, a novel virus, vB_TgeS_JQ, infecting Flavobacteriia was isolated from the surface water of the Golden Beach of Qingdao, China. Transmission electron microscopy demonstrated that vB_TgeS_JQ had the morphology of siphovirus. The experiments showed that it was stable from −20°C to 45°C and pH 5 to pH 8, with latent and burst periods both lasting for 20 min. Genomic analysis showed that the phage vB_TgeS_JQ contained a 40,712-bp dsDNA genome with a GC content of 30.70%, encoding 74 open-reading frames. Four putative auxiliary metabolic genes were identified, encoding electron transfer-flavoprotein dehydrogenase, calcineurin-like phosphoesterase, phosphoribosyl-ATP pyrophosphohydrolase, and TOPRIM nucleotidyl hydrolase. The abundance of phage vB_TgeS_JQ was higher during *Ulva prolifera* (*U. prolifera*) blooms compared with other marine environments. The phylogenetic and comparative genomic analyses revealed that vB_TgeS_JQ exhibited significant differences from all other phage isolates in the databases and therefore was classified as an undiscovered viral family, named *Zblingviridae*. In summary, this study expands the knowledge about the genomic, phylogenetic diversity and distribution of flavobacterial phages (flavophages), especially their roles during *U. prolifera* blooms.

**IMPORTANCE:**

The phage vB_TgeS_JQ was the first flavobacterial phage isolated during green tide, representing a new family in *Caudoviricetes* and named *Zblingviridae*. The abundance of phage vB_TgeS_JQ was higher during the *Ulva prolifera* blooms. This study provides insights into the genomic, phylogenetic diversity, and distribution of flavophages, especially their roles during *U. prolifera* blooms.

## INTRODUCTION

Viruses are the most abundant and diverse “biological entities” in the diverse marine environments and play a crucial role in controlling microbial communities (1, 2). In the surface sea, the abundance of bacteriophages is about 10 times of their host cells, and they can infect and lysis about 30% of bacterial cells every day (3, 4). Phage reproduction mainly depends on the heterotrophic and autotrophic prokaryotes (5). Some phages regulate the biogeochemical cycle of marine bacteria and promote the evolution of marine bacteria by altering their genomes (6, 7). The putative auxiliary metabolic genes (AMGs) expressed by phage can regulate the metabolic pathway of host cells and improve the adaptability of phage (8, 9).

The metagenomic study of viruses has contributed to the understanding of the community structure and function of marine viruses (10). However, most viruses cannot be categorized in the virus community database currently (11). The isolation and genome analysis of phage strain could provide very important linkages between uncultured viral genomes from metagenomes and their potential hosts and on the ecology and genetic evolution of marine viruses (12). However, the phage isolates are still quite few comparing with the prokaryotic isolates.

Green tides are mainly caused by the massive accumulation of macroalgae *Ulva prolifera*, which has been increasing globally in recent years (13). The decomposition of the *U. prolifera* consumes a lot of oxygen and releases hydrogen sulfide, which leads to coastal hypoxia and acidification (14). Furthermore, the degradation of *U. prolifera* generates large amounts of dissolved organic matter (DOM) (15), which exerts a considerable influence on the structure of microbial communities (16). Such large-scale algal blooms may have an impact on marine ecosystems, affecting water quality and the survival and reproduction of marine organisms (13). Therefore, green tide is an important ecological event in marine ecosystems and is closely related to the marine environment. *Flavobacteriia* are a major clade of the *Bacteroides* in the marine environment that dominates during algal blooms, including the largest green tide caused by *U. prolifera* (17), and are heterotrophic bacteria that target complex organic matter and specialize in degrading organic polymers (18). They can decompose complex organic matter by direct attachment and disruption of the glycosidic bonds of polysaccharides by exogenous enzymes to maximize the available degradation of the complex structure of polysaccharides (19). Seaweed polysaccharides are diverse and chemically complex and are broken down by abundant carbohydrate-active enzymes in the marine biochemical cycle, forming one of the largest carbon cycles in the coastal seas. The largest pool of algal lyase genes, namely, CAZymes, resides in the marine ecosystem itself (20), with the main microbial source of the CAZymes gene pool from marine *Flavobacteriia*.

Although *Flavobacteriia* play important roles in the ocean, few flavophages have been isolated. These flavophages contain polysaccharide-degrading enzymes that cleave bacterial polysaccharides such as peptidoglycan and chitin (21). They could regulate carbon cycling in coastal shelf waters by cracking the dynamics of host populations. As the main inhibitors of high-molecular weight algal derivatives, flavophages have been poorly studied (22). Several *Cellulophaga* phages were reportedly isolated in the Baltic Sea, belonging to the *Duplodnaviria* and *Monodnaviria* realms (23). *Tenacibaculum* phage pT24 was isolated from the water of *Penaeus vannamei* aquaculture ponds in Thailand and can infect both *Tenacibaculum mesophilum* and *Tenacibaculum discolor* (24). PTm1 and PTm5, both found in seawater near aquaculture farms in Japan, can infect *Tenacibaculum maritimum*, which can cause multiple outbreaks of farmed marine ichthyosis worldwide (25, 26). Recently, researchers used a culture method to obtain 44 flavophages, representing 12 new species from two viral realms (27). Currently, only 65 complete genomes of flavophages have been uploaded to GenBank (Table 1). Their genomes are highly variable in size (6.6–124.2 kbp), and they contain low GC content (28.7%–38.1%). Although they both belong to flavophages, their hosts are different (Table 1). In consideration of the ecological significance of *Flavobacteriia*, the diversity of flavophages remains low. Additionally, there have been no reports of isolated flavophages during the *U. prolifera* blooms so far.

**TABLE 1:** The genome information of Flavobacteria phage deposited to the GenBank

Phage name	Phage genus	Host bacteria	Genome size (kbp)	GC content (%)	ORFs	tRNA genes	Accession	Number of strains
vB_TgeS_JQ^*a*^	*Zytvirus*	*Tenacibaculum*	40.7	30.7	74	0	MT002873	1
Molly; Colly^*b*^	*Mollyvirus*	*Maribacter*	124.2	36.2	193–201	0	MT732450	7
Gundel^*b*^	*Gundelvirus*	*Tenacibaculum*	78.5	30.4	118	10	MT732474	1
Calle^*b*^	*Callevirus*	*Cellulophaga*	73.0	38.1	85–86	20	MT732432	3
Nekkels^*b*^	*Nekkelsvirus*	*Cellulophaga*	53.3–54.3	31.5	93–94	0	MT732435	2
Omtje^*b*^	*Omtjevirus*	*Cellulophaga*	6.6	31.2	13	0	MT732445	5
Ingeline^*b*^	*Ingelinevirus*	*Cellulophaga*	42.6	32.2	49–50	0	MT732435	8
Leef^*b*^	*Leefvirus*	*Polaribacter*	37.5	29.7	48	0	MT732473	1
Danklef^*b*^	*Freyavirus*	*Polaribacter*	47.2–48.2	28.9	76–78	1	MT732458	5
Freya^*b*^	*Freyavirus*	*Polaribacter*	44.0–48.9	28.9	66–78	0	MT732463	10
Peternella^*b*^	*Peternellavirus*	*Winogradskyella*	39.6	35.3	62	0	MT732475	1
Harreka^*b*^	*Harrekavirus*	*Olleya*	43.2	32.0	80	0	MT732457	1
pT24^*b*^	*Kungbxnavirus*	*Tenacibaculum*	23.4	28.7	297	0	NC_049383	1
PTm1^*b*^	*Shirahamavirus*	*Tenacibaculum*	22.4	29.7	104	0	NC_049340	1
PTm5^*b*^	*Shirahamavirus*	*Tenacibaculum*	22.6	29.7	96	0	AP019525	1
Larrie^*b*^	*Cbastvirus*	*Tenacibaculum*	77.5	33.7	111	0	ON950054	1
vB_FspS_snusmum6-1^*b*^	*Muminvirus*	*Flavobacterium*	39.3	29.1	72	0	NC_048841	1
vB_FspS_mymlan6-1^*b*^	*Muminvirus*	*Flavobacterium*	38.5	29.1	68	0	NC_048838	1
vB_FspS_mumin9-1^*b*^	*Muminvirus*	*Flavobacterium*	38.5	29.1	67	0	NC_048837	1
vB_FspS_hattifnatt9-1^*b*^	*Hattifnattvirus*	*Flavobacterium*	39.2	29.2	76	0	NC_048832	1
vB_FspS_tooticki6-1^*b*^	*Muminvirus*	*Flavobacterium*	36.9	29.4	65	0	NC_048844	1
vB_FspS_filifjonk9-1^*b*^	*Muminvirus*	*Flavobacterium*	38.0	29.3	63	0	NC_048831	1
vB_FspS_morran9-1^*b*^	*Lillamyvirus*	*Flavobacterium*	38.4	29.2	64	0	NC_048836	1
vB_FspS_lillamy9-1^*b*^	*Lillamyvirus*	*Flavobacterium*	38.3	29.1	73	0	NC_048835	1
vB_FspS_stinky9-1^*b*^	*Lillamyvirus*	*Flavobacterium*	38.5	29.3	69	0	NC_048842	1
vB_FspS_hemulen6-1^*b*^	*Lillamyvirus*	*Flavobacterium*	39.0	29.4	72	0	NC_048833	1
vB_FspS_snork6-1^*b*^	*Lillamyvirus*	*Flavobacterium*	38.2	29.5	71	0	NC_048840	1
vB_FspS_sniff9-1^*b*^	*Lillamyvirus*	*Flavobacterium*	38.1	29.4	73	0	NC_048839	1

^*a*^ Phage isolated in this study.

^*b*^ Flavobacteria phages: the genome sequences were down loaded from the NCBI database.

To fill this gap, this study isolated and identified a novel flavophage, named vB_TgeS_JQ, during the *U. prolifera* blooms. This was important for studying the interaction and coevolution between phages and their hosts and undoubtedly an important complement to this phage family. It will be of great significance to further study the contribution of ﬂavophages in the biogeochemical cycle and controlling the abundance, diversity, and evolution of microorganisms in the future.

## MATERIALS AND METHODS

### Sampling

Surface seawater samples were collected from the Golden Beach Sea Area of Qingdao, China (35.57°N, 120.14°E), on 24 July 2019 and stored at 4°C. From June to July, a large-scale *U. prolifera* bloom occurs in Yellow Sea since 2007. In 2019, the total coverage area around Qingdao reached up to 195 km^2^ (https://www.gov.cn/xinwen/2019-08/06/content_5419215.htm#1).

### Bacterial strain isolation, purification, and identification

The seawater samples were diluted with Zobell 2216E medium in gradient (0 to 10^−5^), and then, 200 µL liquid was inoculated and incubated on a 2216E solid plate. Each gradient was parallel in three groups and placed in an incubator at 28°C and 120 rpm for 12 h. Then, 20 to 30 single colonies with distinct morphological characteristics were selected and placed on the new plates in three areas, respectively. This bacterial strain was purified three to five times until a pure single colony was obtained and stored in the refrigerator at 4°C. Then, the 16s rRNA of the bacterial strain was amplified by PCR and sequenced, and BLASTn search was performed to determine the host taxonomic information.

### Phage isolation, purification and concentration

The seawater samples were filtered through the 0.22-µm pore-size membranes (Millipore) to remove bacteria and phytoplankton. Then, the phage plaques were isolated by the double-layer agar method (28). In brief, 200 µL of seawater samples was mixed with 200 µL of logarithmic growth phase bacteria and incubated at 25°C for 15 min. The Zobell 2216E semisolid medium (45°C) was vortexed with the above mixture and poured on the surface of the solid medium. After overnight incubation at 28°C, the formation of plaques was observed. The single plaque was picked out, suspended in 1 mL of SM buffer (100 mM NaCl, 81.2 mM MgSO4.7 H_2_O, 50 mM Tris-HCl, pH = 7.5), and incubated for 1 h at 37°C. Then, the mixture was filtered onto a 0.22-mm PES Millipore filter to get the phage suspension; the double-layer agar method was repeated 3–5 times to ensure that the phage solution was completely purified. The purified phage solution was expanded to 500 mL and incubated with 10% (wt/vol) PEG8000 at 4°C for 24 h in the dark. Then, bacteria were removed by filtrating viral cultures with a 0.22-µm membrane filter (Millipore). The solution was centrifuged at 10,000 *g* at 4°C for 30–45 min, and only the precipitate was retained. Then the precipitate was resuspended in 5 mL SM buffer and stored in SM buffer at 4°C until treatment (29).

### Transmission electron microscopy

The morphology of vB_TgeS_JQ was observed by transmission electron microscopy (TEM). A volume of 20 µL of the enriched phage suspension was negatively stained with phosphotungstic acid (2% wt/vol, pH 7.2) for 15 min (30). The morphology was observed under a transmission electron microscope (HT7700 Exalens, Japan) at 100 kV to determine the structural features (31). The bar represents 100 nanometers.

### One-step growth curve, thermal, and pH tolerance analyses of phage vB_TgeS_JQ

One-step growth curve is a common experimental procedure for studying phage propagation (32). In summary, 1 mL of exponential growth phase (2.0 × 10^8^ CFU/mL) host bacteria was mixed with an equal volume of the phage vB_TgeS_JQ (multiplicity of infection = 0.1) and incubated at 28°C for 15 min. Then, 1 mL mixture was centrifuged at 13,000 *g* for 1 min to remove the upper liquid and 1 mL of Zobell 2216E culture medium was added to briefly vortex. This step was repeated three times to remove unabsorbed phages. Then, the sample was added to 300 mL 2216E medium to suspend the cells and shaken at 28°C for 80 min. Samples were taken with 5-min intervals and then were coated on double plates and incubated overnight after collection. The number of plaques was counted, phage titer was calculated at different periods, and phage growth status was determined.

Then, to analyze the tolerance of phage vB_TgeS_JQ to temperature, the 200-µL phage samples (10^8^ PFU/mL; pH = 7.0) were placed at −20°C, 4°C, 25°C, 35°C, 45°C, 55°C, 65°C, and 75°C for 2 h. Until the temperature returned to room temperature, 200-µL host bacteria solution was added to infect phage vB_TgeS_JQ for 15 min, and then, the plaque was detected using a double plate method. All analyses were performed in triplicate.

Nine hundred microliters of SM buffer with pH (3–12) was added to each 100 µL phage sample (10^8^ PFU/mL). The 200-µL samples of different pH gradients were mixed with an equal volume of host bacteria solution, and the double plate was poured after 15 min. All analyses were performed in triplicate. After culturing at 28°C for 12 h, the influence trend of pH was calculated and plotted.

### Phage DNA extraction, sequencing, and bioinformatics analysis

Viral genomic DNA extraction was performed by the HP Viral DNA Kit (OMEGA) according to the instructions. Purified DNA samples were sequenced using Illumina NovaSeq PE150 paired-end method. GapCloser v1.12 was used to close the gaps between the remaining contigs using purified genomic DNA as a template. Clean data were obtained by filtering raw data and assembled with SOAP *de novo* v2.04 (33). The sequences of the assembled phages were then used to predict open-reading frames (ORFs) with RAST (http://rast.nmpdr.org/) and GeneMarks (http://topaz.gatech.edu/GeneMark/). tRNAs were predicted by the tRNAscan-SE program (http://lowelab.ucsc.edu/tRNAscan-SE/) (34). The annotation of ORFs was predicted by BLASTp (*E* value < 1*e*^−10^) (http://blastp.ncbi.nlm.nih.gov/), a Pfam search with default parameters (https://pfam.xfam.org/search/sequence), and online server for HHpred search (*E* value ≤ 10^−3^) (https://toolkit.tuebingen.mpg.de/hhpred) (35–37). Genomic mapping was performed using R (38).

### Taxonomic and phylogenetic analysis

vConTACT (version 2.0) (39) was performed to explore the accurate taxonomic position of phage vB_TgeS_JQ and its homology with other phages. The sequence of vB_TgeS_JQ was used as a query to search the NCBI database with tBLASTx (*E* value ≤ 1*e*^−5^, query coverage ≥ 50%, and amino acid identity ≥ 30%) and the Integrated Microbial Genomes/Virus 4.0 (IMG/VR4.0) database with DIAMOND BLASTp (*E* value ≤ 1*e*^−10^, query coverage ≥ 50%, and amino acid identity ≥ 30%). Viral clusters were identified using ClusterONE with default parameters defined in vConTACT 2.0. The network visualization based on vConTACT 2.0 analysis was conducted by Gephi (40).

According to the results of vConTACT 2.0, ViPTree (https://www.genome. jp/viptree) (41) was used to determine the taxonomic position of vB_TgeS_JQ. The genome sequences closely related to vB_TgeS_JQ were selected to draw a rectangular proteomic tree using tBLASTx and VipTree (42). The phylogenetic tree based on the viral conserved proteins (major capsid protein, terminase large subunit, and portal protein) was constructed to evaluate the evolutionary relationship between vB_TgeS_JQ and other diverse phages. Sequence alignment was performed with MUSCLE (version 91), followed by running IQtree 2.0 (version 92) (43) with a bootstrap of 1,000. The treefile generated by GBDP_Trimming_D6_FASTME was selected, and the visualization was performed using iTol (version 5) (44).

Average Nucleotide Identity (ANI) was obtained using VIRIDIC (version 1) to determine the overall similarity between two genomic sequences and then visualized by pheatmap in R.

All-to-all BLASTP analysis was performed by OrthoFinder (version 2.5.4) to calculate the percentage of shared homologous protein-coding genes between phage vB_TgeS_JQ and its closest relatives (*E* value < 1*e*^−5^, query cover > 50%, and identity > 30%).

### Ecological distribution in the ocean

The relative abundances of vB_TgeS_JQ were calculated using CoverM v0.6.1 (https://github.com/wwood/CoverM) and expressed by RPKM (reads per kilobase per million mapped reads) (v0.3.1) with parameters: -m rpkm--min-read-percent-identity 0.95--min-read-aligned-percent 0.75 (45, 46). The quality-controlled reads of 154 Global Ocean Viromes (GOV 2.0) and Qingdao coastal virome (QDCV) data sets (47) were mapped to the vB_TgeS_JQ genome using metagenomics tool minimap2. QDCV data set was selected because the phage was isolated during an outbreak of *U. prolifera* in coastal Qingdao, during which *Flavobacteriia* was the dominant strain. The GOV 2.0 data set defined five marine viral ecological zones (VEZs) as Arctic (ARC), Antarctic (ANT), temperate and tropical epipelagic (EPI), temperate and tropical mesopelagic (MES), and bathypelagic (BATHY) (11). Several phages with higher abundance in the ocean were selected as references, including representative pelagibacter phages (HTVC010P, HTVC019P, HTVC011P, and HTVC008M), cyanophages (P-SSB7, P-SSM7, and S-RIM4) (48), *Synechococcus* phages (S-ShM2, S-SSM5, P60, S-B28, and S-B68), *Prochlorococcus* phages (P-HM1, P-HM2, P-RSM4, P-SSM2, and P-SSM4), and some closely related flavophages (vB_FspS_snusmum6-1, vB_FspS_snusmum6-1, vB_FspS_mumin9-1, vB_FspS_hattifnatt9-1, vB_FspS_tooticki6-1, and vB_S_filifjonk9-1), as well as four non-redundant homology uncultured viral genomes (UViGs) taxonomically associated with vB_TgeS_JQ. We also calculated the relative abundances of vB_TgeS_JQ and its closely related flavophages based on several virus metagenomic data during outbreak and the extinction process of *U. prolifera* in coastal Qingdao in 2020 (49).

### Genome sequence accession number

The annotation results and related information were uploaded to GenBank to obtain the accession number. The complete genome sequence of phage vB_TgeS_JQ is available in the GenBank database under accession number MT002873.

## RESULTS AND DISCUSSION

### Morphology and characterization of vB_TgeS_JQ

**Fig 1:**
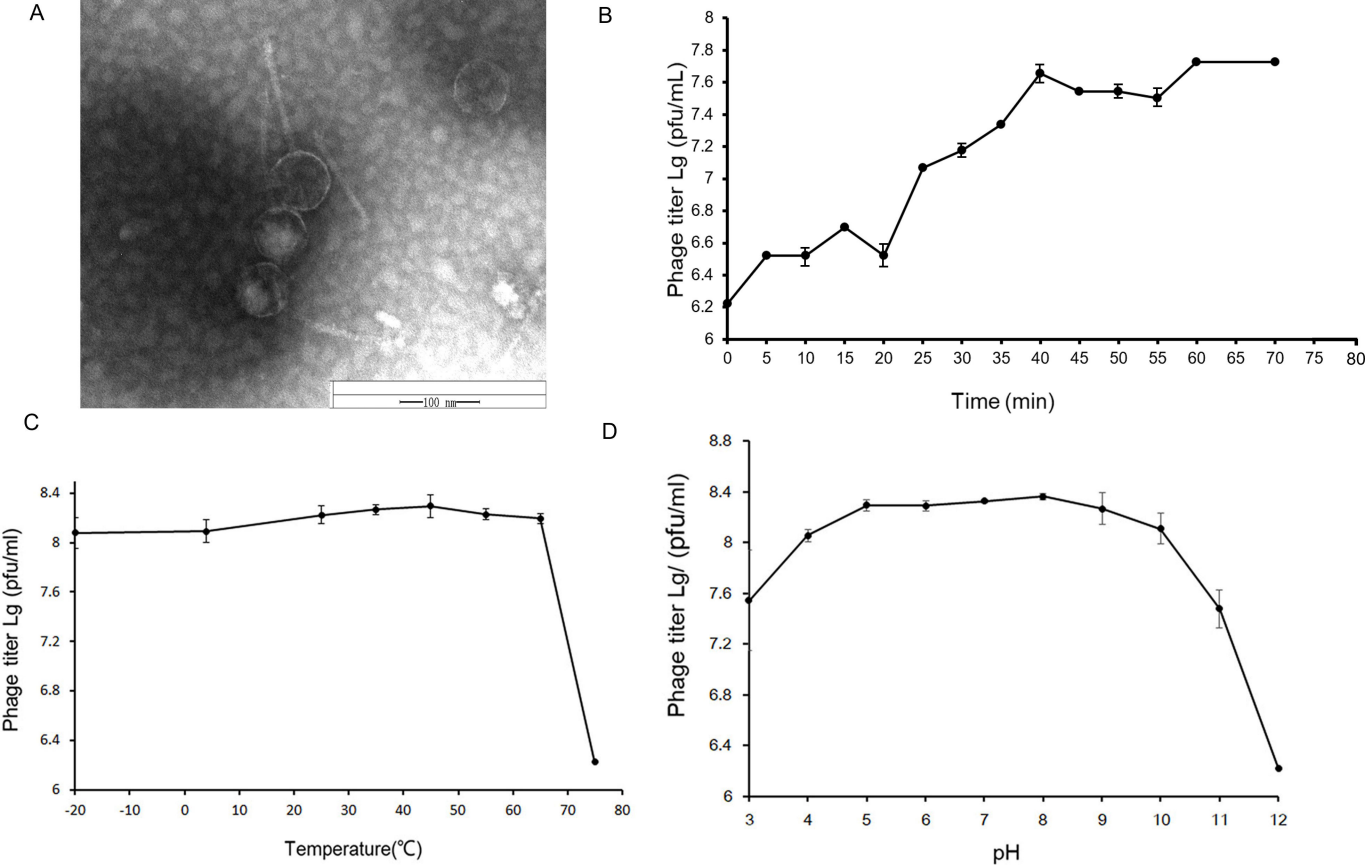
(**A**) TEM of phage vB_TgeS_JQ. The scale bar is 100 nm. (**B**) One-step growth curve of vB_TgeS_JQ. The data shown are average values from triplicate experiments, and error bars indicate standard deviations (SDs). (**C**) The curve of thermal stability of vB_TgeS_JQ. (**D**) pH stability curve of phage vB_TgeS_JQ. The data shown are average values from triplicate experiments, and error bars indicate SDs. The Y-axis of the ﬁgure is a log scale.

The phage vB_TgeS_JQ was isolated from the Golden Beach Sea Area of Qingdao, China (35.57°N, 120.14°E), regarding *Tenacibaculum geojense* YCS-6 as the host, which belonged to *Flavobacteriia*. The phage vB_TgeS_JQ formed a 0.42-mm needle-eye transparent spot (Fig. 1A), which was densely distributed in double-layer agar. By TEM analysis, the phage contained an icosahedral head (average diameter about 59 nm) and a long tail that cannot retract (average diameter about 103 nm) (Fig. 1A). Based on the morphological characteristics, it was proposed that phage vB_TgeS_JQ belonged to siphovirus.

The characteristics of vB_TgeS_JQ were studied by one-step growth curve, temperature, and pH stability. The results of one-step growth curve (Fig. 1B) showed that the latent period of phage vB_TgeS_JQ was about 20 min. After 20 min, the titer of phage vB_TgeS_JQ showed a significant increase and entered the burst phase. After 40 min, the growth rate of vB_TgeS_JQ was slow and reached a plateau. Approximately 281 virions were released from each host cell (phage particle number at the end of the outbreak: 4.5 × 10^8^ PFU/mL; the initial phage concentration of 1.6 × 10^6^ PFU/mL).

The phage showed the maximum titer at 45°C in the temperature stability experiment. The titer of vB_TgeS_JQ was at a high level when the temperature ranged from −20°C to 45°C (Fig. 1C); however, the phage titer decreased linearly at more than 65°C. In general, vB_TgeS_JQ is more sensitive to high temperature than low temperature, but the optimal growth temperature is concentrated around 45°C. This characteristic may be the result of adaptation to the complex marine environment.

In the pH sensitivity experiment (Fig. 1D), phage vB_TgeS_JQ was stable from pH 5 to 8. The titer of phage vB_TgeS_JQ decreased with the increase of pH at pH 9 to 12, and the decrease was greater than that in acidic environment pH 3 to 5. This result indicated that vB_TgeS_JQ could survive in both acid and base environments but it was more adaptable to acidic environments.

### Overall genome analysis of vB_TgeS_JQ

**Fig 2:**
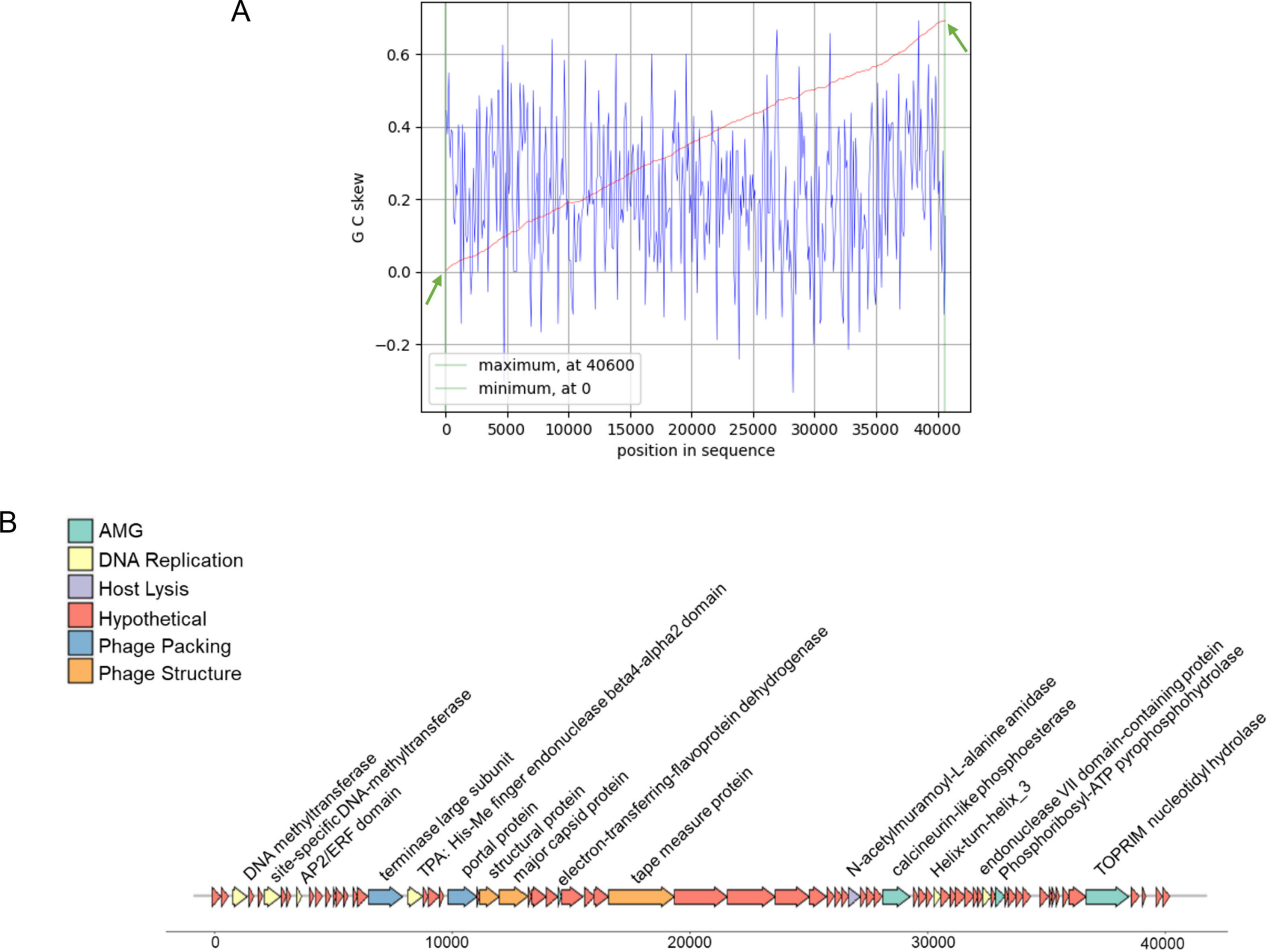
(**A**) The global minimum and maximum values are shown in the cumulative graph and were calculated using a window size of 100 bp and a step size of 100 bp. The GC-skew and the cumulative GC-skew are represented by blue and red lines, respectively. The minimum and maximum values of GC-skew are indicated by two green arrows and could predict the origin and terminal positions of genome replication. (**B**) Genome map and functional protein annotation of the phage vB_TgeS_JQ. Putative function categories were defined according to annotation and are represented by different colors. Each arrow represents the gene length and its transcriptional direction.

According to the sequencing result, the length of putative double-stranded DNA genome of phage vB_TgeS_JQ was 40,712 bp, and the GC content was 30.70%. The cumulative GC skew indicated the predicted origin and terminal positions located at 0 nt and 40,600 nt, respectively (Fig. 2A). A total of 74 ORFs were predicted, 58 (78.4%) of which had no homologous proteins in current public databases called hypothetical proteins and 16 (21.6%) of which had orthologs with known functional genes (Table 2). Based on their functions, 16 ORFs with annotated protein information can be divided into four modules: DNA replication, regulation, and nucleotide metabolism; phage structure; phage packing; and host lysis; four putative AMGs (ORF30, ORF46, ORF59, and ORF70) were also predicted (Fig. 2B).

**TABLE 2:** Protein functional annotation of phage vB_TgeS_JQ

ORF	Start	Stop	Strand	Function	*E* value	Accession
3	866	1,486	+	DNA methyltransferase	8.0*E*^−88^	NRA92623.1 (BLASTp)
6	2,224	2,889	+	Site-specific DNA-methyltransferase	8.0*E*^−108^	WP_051957571.1 (BLASTp)
9	3,591	3,803	+	AP2/ERF domain	3.0*E*^−02^	IPR036955 (Pfam)
18	6,636	8,087	+	Terminase large subunit	4.0*E*^−56^	CAB4128504.1 (HHpred)
19	8,285	8,929	+	TPA: His-Me finger endonuclease beta4-alpha2 domain	4.0*E*^−05^	DAF73219.1 (BLASTp)
23	9,989	11,221	+	Portal protein	4.0*E*^−58^	WP_159288166.1 (BLASTp)
25	11,348	12,175	+	Structural protein	1.0*E*^−101^	YP_008241780.1 (BLASTp)
26	12,180	13,385	+	Major capsid protein	1.0*E*^−163^	WP_055413133.1 (BLASTp)
30	14,656	14,814	+	Electron-transferring-flavoprotein dehydrogenase	2.0*E*^−135^	AEH89775.1 (BLASTp)
34	16,805	19,585	+	Tape measure protein	2.0*E*^−73^	YP_010356880.1 (BLASTp)
42	26,988	27,506	+	N-Acetylmuramoyl-L-alanine amidase	2.0*E*^−19^	WP_005908864.1 (BLASTp)
46	28,418	29,608	+	Calcineurin-like phosphoesterase	3.0*E*^−123^	YP_010356918.1 (BLASTp)
50	30,593	30,898	+	Helix-turn-helix_3	9.1*E*^−07^	PF01381.2 (Pfam)
57	32,663	33,043	+	Endonuclease VII domain-containing protein	1.0*E*^−07^	WP_138650584.1 (BLASTp)
59	33,221	33,604	+	Phosphoribosyl-ATP pyrophosphohydrolase	1.9*E*^−18^	PF01503.20 (Pfam)
70	37,068	38,870	+	TOPRIM nucleotidyl hydrolase	2.0*E*^−175^	CAB5218073.1 (HHpred)

The results indicated that all ORFs were encoded on the positive strand, which showed a higher degree of complementarity with the promoter sequence. Transcription enzymes are easier to recognize and start transcription, which may improve the replication efficiency of the virus in the coastal environment of *U. prolifera* (50).

In the phage vB_TgeS_JQ genome, most ORFs are associated with DNA replication, regulation, and nucleotide metabolism. Both ORF3 and ORF6 encode methyltransferases, which regulate the phage life cycle by affecting the expression of phage genes (51). Many bacteria have a set of self-protection mechanisms, including the CRISPR-Cas system and restriction enzyme system (52). In order to evade the host defense system, some phages will undergo methylation modification on their DNA, which can help them evade the bacterial defense response and better replicate and survive (53). Interestingly, ORF50 encodes the Helix-turn-helix domain, a common DNA-binding domain found in many transcription factors and regulatory proteins, which plays a key role in viruses (54). It can bind to specific DNA sequences in the viral genome and regulate key processes such as transcription, replication, and the life cycle of the virus.

A total of three ORFs were detected to be related to the structure of vB_TgeS_JQ phage. In particular, the genome contained a structural protein called tape measure protein (TMP; ORF30). This protein is an important component of the phage structure and plays a key role in phage assembly and infection (55). TMP can help phage deliver its genome into the cytoplasm of host cells by interacting with cell surface receptors, which is essential for the successful infection of host cells (56).

Two genes associated with vB_TgeS_JQ genome packaging were identified as terminase large subunit (ORF18; TerL) and portal protein (ORF23). TerL recognizes and binds specific sequences of the phage genome and transforms the genome from a chaotic state in the cytoplasm to a tightly packed structure so that it can accommodate the size and shape of the phage head and acts as an endonuclease to cleave viral genomes from the linker (57, 58). Portal protein is an important component of phage structure and plays a key role in the process of phage infection and replication. It plays multiple important roles during phage infection and replication, including injection and release of the phage genome and maintenance of head structural integrity and as localization markers for replication. These functions are important for phage to successfully infect host cells and replicate its own genome (59).

In the phage vB_TgeS_JQ genome lysis module, ORF42 encodes the N-acetylmuramyl-L-alanine amidase, an endotoxin that plays an important role in phages. Its main function is to hydrolyze the N-acetyl muramyl-L-alanine peptide bond on the bacterial cell wall, leading to cell wall degradation and rupture. This enzyme is essential in the process of phage parasitize, contributing to the release of the phage genome and infection of the host bacteria. N-Acetylmuramyl-l-alanine amidase completes the phage life cycle by degrading the bacterial cell wall and facilitating the injection and replication of phage DNA (60).

### Putative AMGs of phage vB_TgeS_JQ

Four putative AMGs were identified in the genome of phage vB_TgeS_JQ, encoding electron transfer-flavoprotein dehydrogenase (ETFDH; ORF30), calcineurin-like phosphoesterase (ORF46), phosphoribosyl-ATP pyrophosphohydrolase (PPAPH; ORF59), and TOPRIM nucleotidyl hydrolase (ORF70), respectively.

In particular, ETFDH is an important enzyme involved in the process of mitochondrial oxidative phosphorylation, which plays a crucial role in intracellular energy metabolism pathways, and is widely present in some bacteria and fungi (61). This enzyme typically catalyzes the transfer of electrons from electron transfer flavoprotein to a terminal electron acceptor such as a quinone or another electron carrier in the respiratory chain. This electron transfer is an essential step in energy metabolism, particularly in processes like beta-oxidation of fatty acids and amino acid metabolism (62). Due to the propagation of U. prolifera, there has been a rise in the number of viruses infecting bacteria. The ETFDH in the vB_TgeS_JQ phage may aid the host *Flavobacteriia* to obtain energy required for cellular metabolism, contributing to its survival chances under coastal conditions.

Calcineurin-like phosphoesterase could regulate the phosphorylation level of intracellular substrates by catalyzing the hydrolysis of phosphate ester bonds, affecting protein synthesis, signal transduction, and cell metabolism (63). Some viruses use host cell phosphatases to regulate intracellular signaling pathways to promote viral replication and spread. In addition, some viruses may encode their own phosphatases, thereby interfering with the signal transduction pathways of host cells, inhibiting the host cellular immune response, or regulating the intracellular environment to promote virus replication.

PPAPH and TOPRIM nucleotide hydrolases are present in many marine phages involved in nucleotide metabolism, which suggests that they have an important role in phage proliferation (64). In the phage vB_TgeS_JQ genome, PPAPH and TOPRIM nucleotide hydrolases may regulate host nucleotide synthesis and utilization by infecting host cells to sustain their own growth and improve survival in coastal environments.

### Phylogenetic and comparative genomic analyses of phage vB_TgeS_JQ

**Fig 3:**
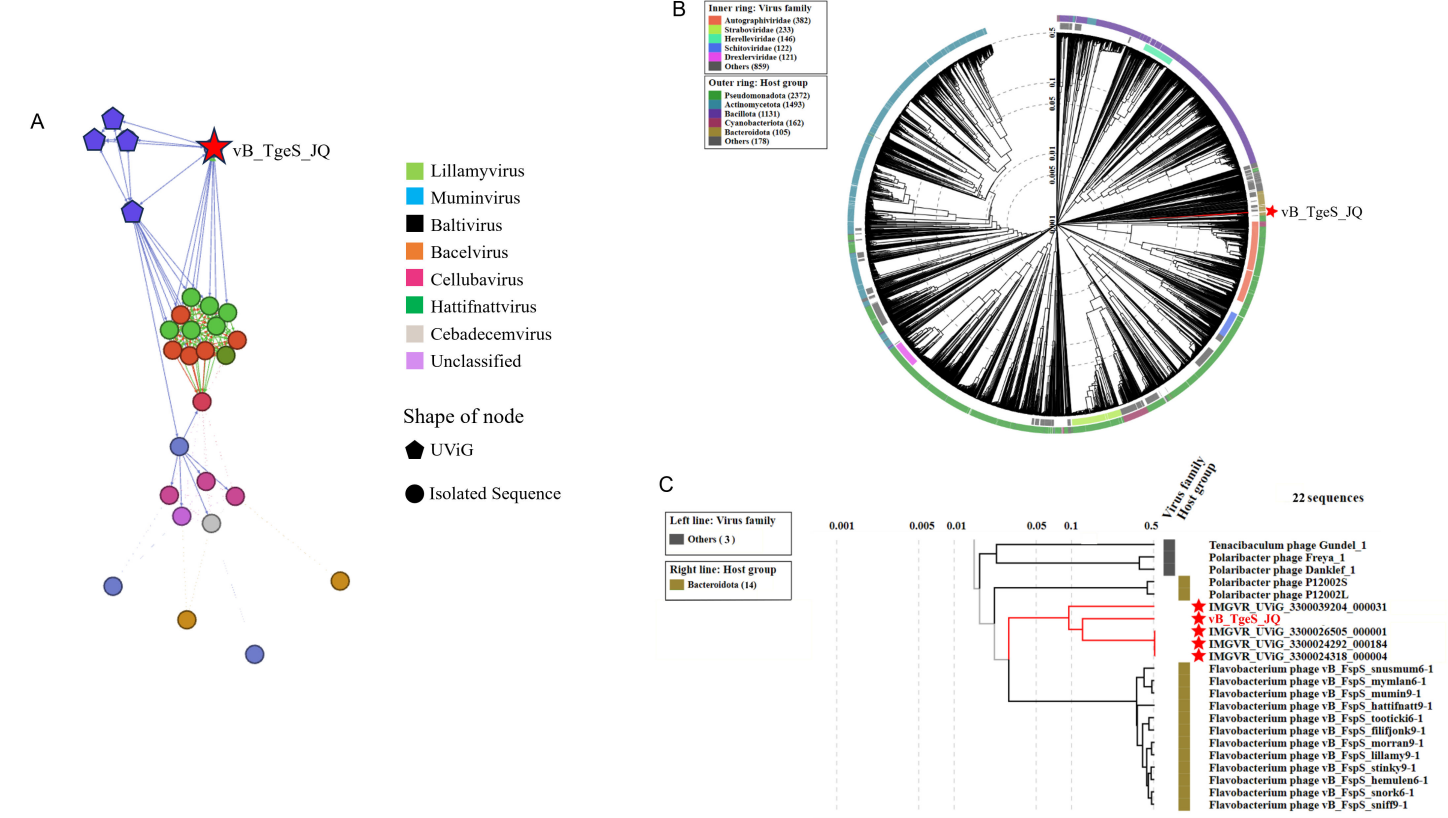
(**A**) Gene content-based viral network among vB_TgeS_JQ and vB_TgeS_JQ-associated genomes from the NCBI virus database and IMG/VR 4.0 database. Nodes represent the viral genomic sequences. Edges represent the similarity scores between genomes based on shared gene content. Regular circles represent isolated viral sequences, and the UViGs from IMG/VR are represented by hexagons. The star represents phage vB_TgeS_JQ. The genomes of viruses belonging to different genera are represented by different colors. (**B**) The circular view of phage vB_TgeS_JQ, constructed using VipTree. The colored rings represent the virus families (inner ring) and host groups (outer ring). The red star represents the position of the phage vB_TgeS_JQ. (**C**) Phylogenetic tree of phage vB_TgeS_JQ and 21 viruses that were closely related to vB_TgeS_JQ. These trees were calculated by BIONJ according to the genome distance matrix and take the midpoint as the root.

According to the search results in the NCBI and the IMG/VR database, only four metagenomic assembled uncultured viral sequences (IMGVR_UViG_3300039204_000031, IMGVR_UViG_3300024318_000004, IMGVR_UViG_3300026505_000001, and IMGVR_UViG_3300024292_000184; *E* value ≤ 10^−5^; query cover ≥ 50%) were matched with phage vB_TgeS_JQ. The network diagram revealed that vB_TgeS_JQ was classified as an outlier related to IMGVR_ UViG_3300024292_000184 and IMGVR_UViG_3300039204_000031 (Fig. 3A).

The whole genome phylogenetic analysis revealed that the genome of phage vB_TgeS_JQ formed a distinct branch separated from other sequences, indicating a potential new viral cluster (Fig. 3B and C).

**Fig 4:**
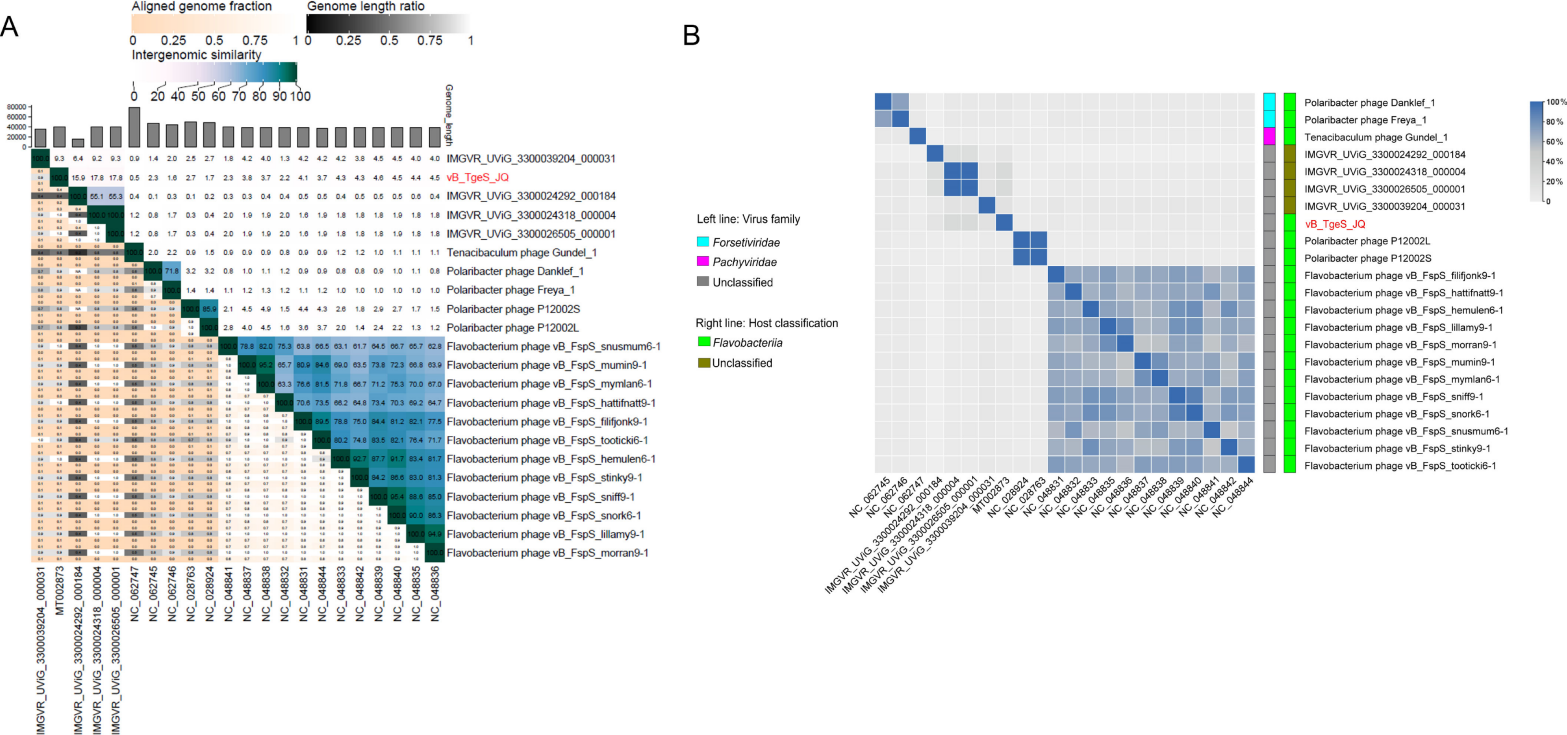
(**A**) Intergenomic similarities between vB_TgeS_JQ and its distantly related phages generated by VIRIDIC. These numbers represent ANI. (**B**) The heat map shows shared genes among vB_TgeS_JQ with flavophages. The ratio of shared genes was based on all-vs-all BLASTp analysis, which was performed by using OrthoFinder with the following parameters: cutoff *E* value < 1*e*^−5^, identity > 30%, and alignment region covering > 50% of the shorter sequence. The cluster method was complete, which defined the class-to-class distance as the complete distance between samples.

**Fig 5:**
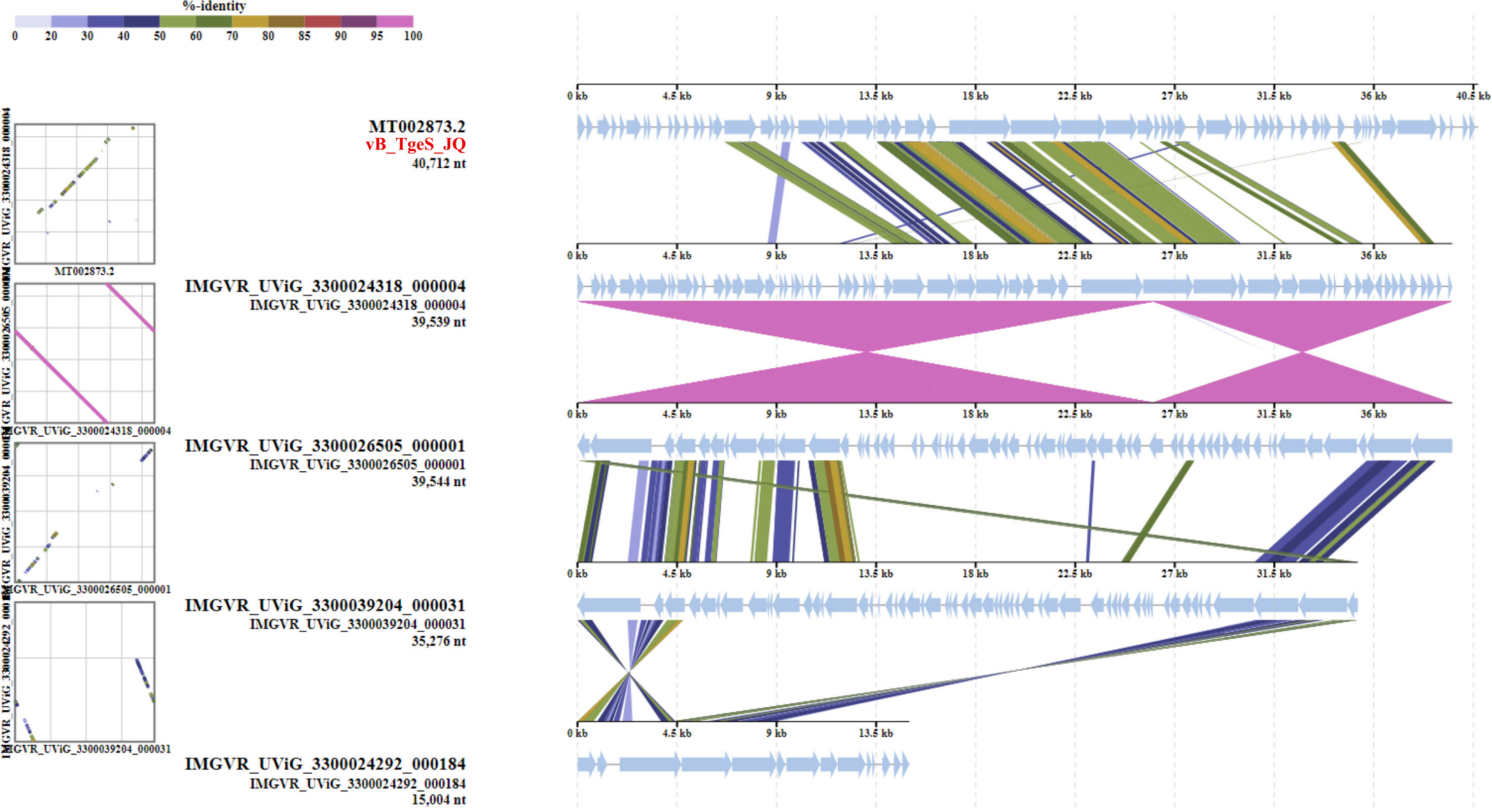
Genomic comparisons between phage vB_TgeS_JQ and four related closely phages. The shading below each genome indicates sequence similarities between the genomes, with different colors representing the levels of similarity.

Then, ANI analysis of the 22 phage genomes in the phylogenetic tree showed low nucleotide identity between vB_TgeS_JQ and other phages, ranging from 0.5% to 17.8% (Fig. 4A). Additionally, all-vs-all BLASTp analysis revealed less than 20% genetic similarity between vB_TgeS_JQ with the other 21 phages (Fig. 4B). According to the definition provided by the ICTV, when the genomic similarity between a phage and other phages is below 50%, it could be classified as a new family. Furthermore, based on these findings, four closely related phages (IMGVR_UViG_3300039204_000031; IMGVR_UViG_3300024318_000004; IMGVR_UViG_3300026505_000001; IMGVR_UViG_3300024292_000184) were selected for comparative genomic analysis (Fig. 5). This analysis confirmed the low similarity of vB_TgeS_JQ with these phages. Consequently, vB_TgeS_JQ could be classified as a representative of an unexplored viral family. To fully verify the reliability of the conclusions, three protein phylogenetic trees were constructed using conserved viral proteins including TerL, MCP, and portal protein. The results showed that phage vB_TgeS_JQ had conserved proteins that are distinct from other phages, forming a new branch in the phylogenetic tree (Fig. S1). These results suggest that vB_TgeS_JQ has the potential to split into a new family within *Caudoviricetes*, named here as *Zblingviridae*.

### Abundance of vB_TgeS_JQ in marine environments

**Fig 6:**
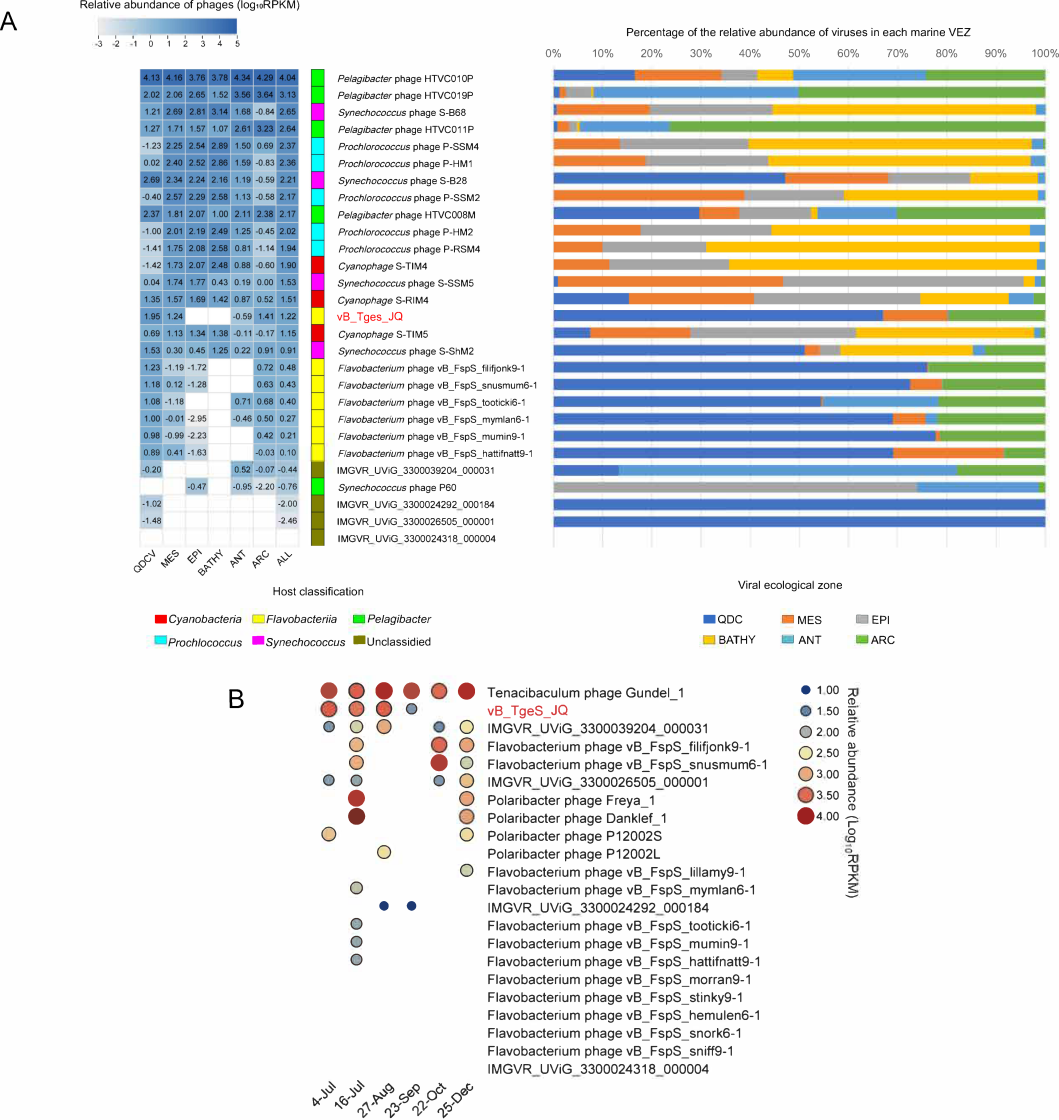
(**A**) Relative abundance of phage vB_TgeS_JQ in marine environments. Relative abundance was calculated using CoverM v0.6.1, which is expressed by RPKM values. Left, the heatmap reflects the relative abundances of the vB_TgeS_JQ and other phages in five marine VEZs defined by the GOV2.0 (ARC, ANT, BATHY, EPI, and MES) and QDCV. Right, the bar graph shows the percentage of bacteriophages in the five VEZs and QDCV. (**B**) Temporal changes of phage vB_TgeS_JQ. Bubble size shows relative abundance on the phage vB_TgeS_JQ of different time series, expressed by RPKM values.

The relative abundance of vB_TgeS_JQ was described in the QDCV and the 154 viral metagenomes from five VEZs of the GOV 2.0 data set: ARC, ANT, EPI, MES, and BATHY (11). The results revealed a significantly higher relative abundance of phage vB_TgeS_JQ in the area where the *U. prolifera* blooms off the coast of Qingdao compared with that of the GOV2.0 data set (Fig. 6A). The relative abundance of flavophages, closely related to phage vB_TgeS_JQ, exhibited a higher occurrence in the QDCV compared with the GOV2.0 data set. The temporal abundances of phage vB_TgeS_JQ and its closely related flavophages were characterized in several virus metagenome data in Qingdao coastal waters in 2020. The results indicated that the relative abundance of phage vB_TgeS_JQ increased in July and August and decreased in September (Fig. 6B). These findings implied that the proliferation of ﬂavophages during the *U. prolifera* period potentially related to a concomitant increase in their host cells. These results indicated the dynamic relationship between viruses and their hosts in coastal ecosystems. The abundance of phage vB_TgeS_JQ at the outbreak of *U. prolifera* may be due to the accumulation of a large number of algae-derived polysaccharides after the arrival of algal blooms (47). *Flavobacteriia* are the dominant prokaryotic group during this green tide period (17, 65), as they can degrade algae-derived polysaccharides (66).

Interestingly, some flavophages had slightly higher abundances in October and December (Fig. 6B). We speculate that this may be due to the higher nutrient concentration at the end of the bloom termination stage (67).

### Conclusion

*Flavobacteriia* are widely recognized as the predominant prokaryotic group during algal blooms and play a crucial role in global biogeochemical cycles. However, only a few flavophages have been isolated and studied, particularly from green tide. In this study, a new flavophage vB_TgeS_JQ was isolated from the coastal of Qingdao, China, representing a new viral family within the *Caudoviricetes*, namely, *Zblingviridae*. The phage vB_TgeS_JQ had a long incubation period and a small burst size. Moreover, the abundance of phage vB_TgeS_JQ was higher during the *U. prolifera* blooms compared with other marine environments, especially during the outbreak period. The four putative AMGs may improve the survival of the host and the phage itself during the *U. prolifera* blooms by affecting the host’s cell metabolism. This study is of great significance to further study the contribution of ﬂavophages in the biogeochemical cycle and controlling the abundance, community, and evolution of microorganisms in the future.

## SUPPLEMENTAL MATERIAL

Sequence S1 aem.00367-24-s0001.txt IMGVR_UViG_3300024318_000004 amino acid sequence.

Sequence S2 aem.00367-24-s0002.txt IMGVR_UViG_3300024292_000184 nucleotide sequence.

Sequence S3 aem.00367-24-s0003.txt IMGVR_UViG_3300026505_000001 nucleotide sequence.

Sequence S4 aem.00367-24-s0004.txt IMGVR_UViG_3300039204_000031 nucleotide sequence.

Fig. S1 aem.00367-24-s0005.tif Phylogenetic trees of marker genes.

Fig. S2 aem.00367-24-s0006.tif Phylogenetic tree of host.

ASM does not own the copyrights to Supplemental Material that may be linked to, or accessed through, an article. The authors have granted ASM a non-exclusive, world-wide license to publish the Supplemental Material files. Please contact the corresponding author directly for reuse.
