# Supplementary figures and images for: A novel flavobacterial phage abundant during green tide, representing a new viral family, Zblingviridae
Source: Appl Environ Microbiol. 2024 Jul 2;90(7):e00367-24. doi: 10.1128/aem.00367-24 (PMC11267871; doi:10.1128/aem.00367-24)

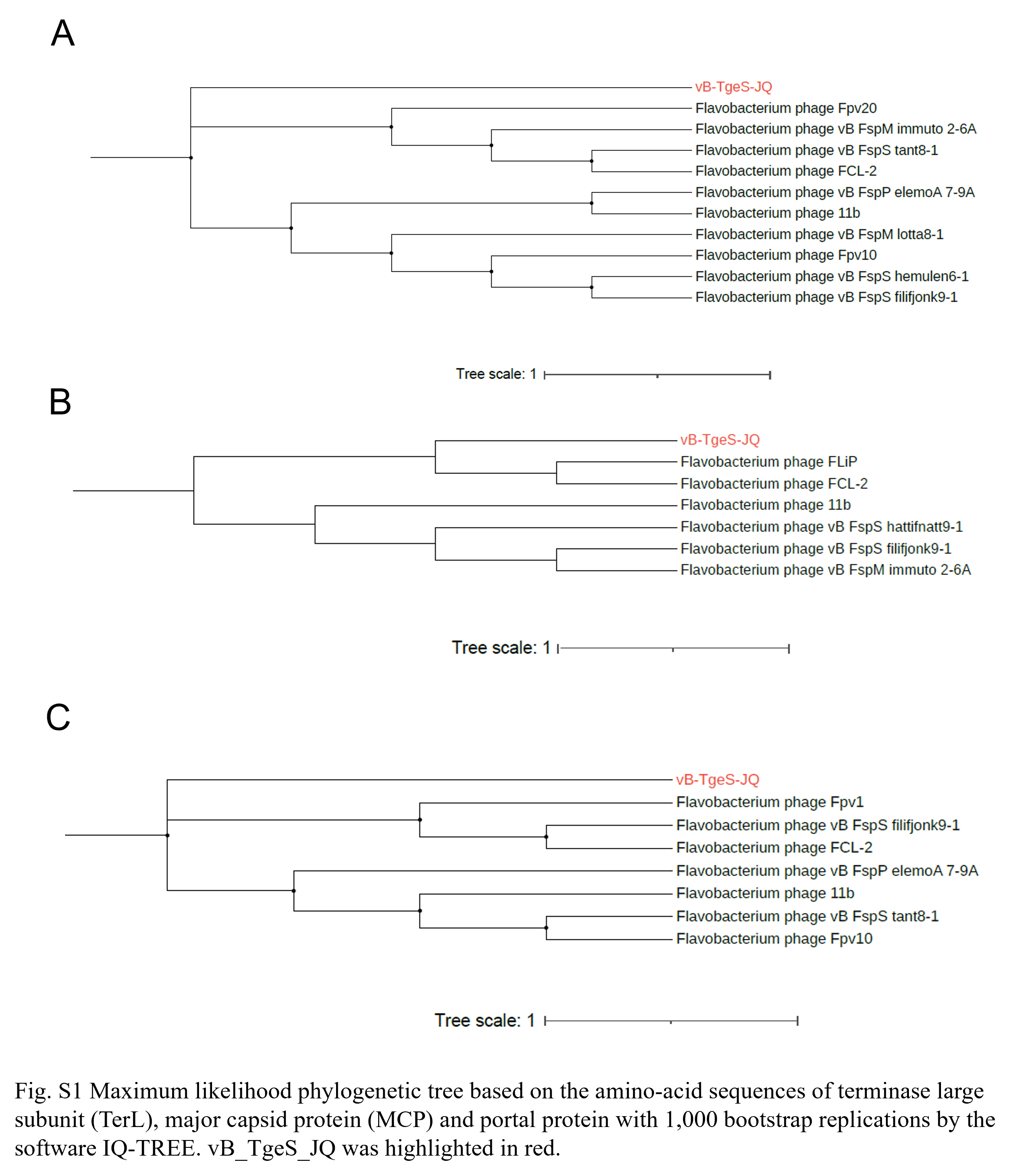

Supplement: Fig. S1 — Phylogenetic trees of marker genes. [file aem.00367-24-s0005.tif]

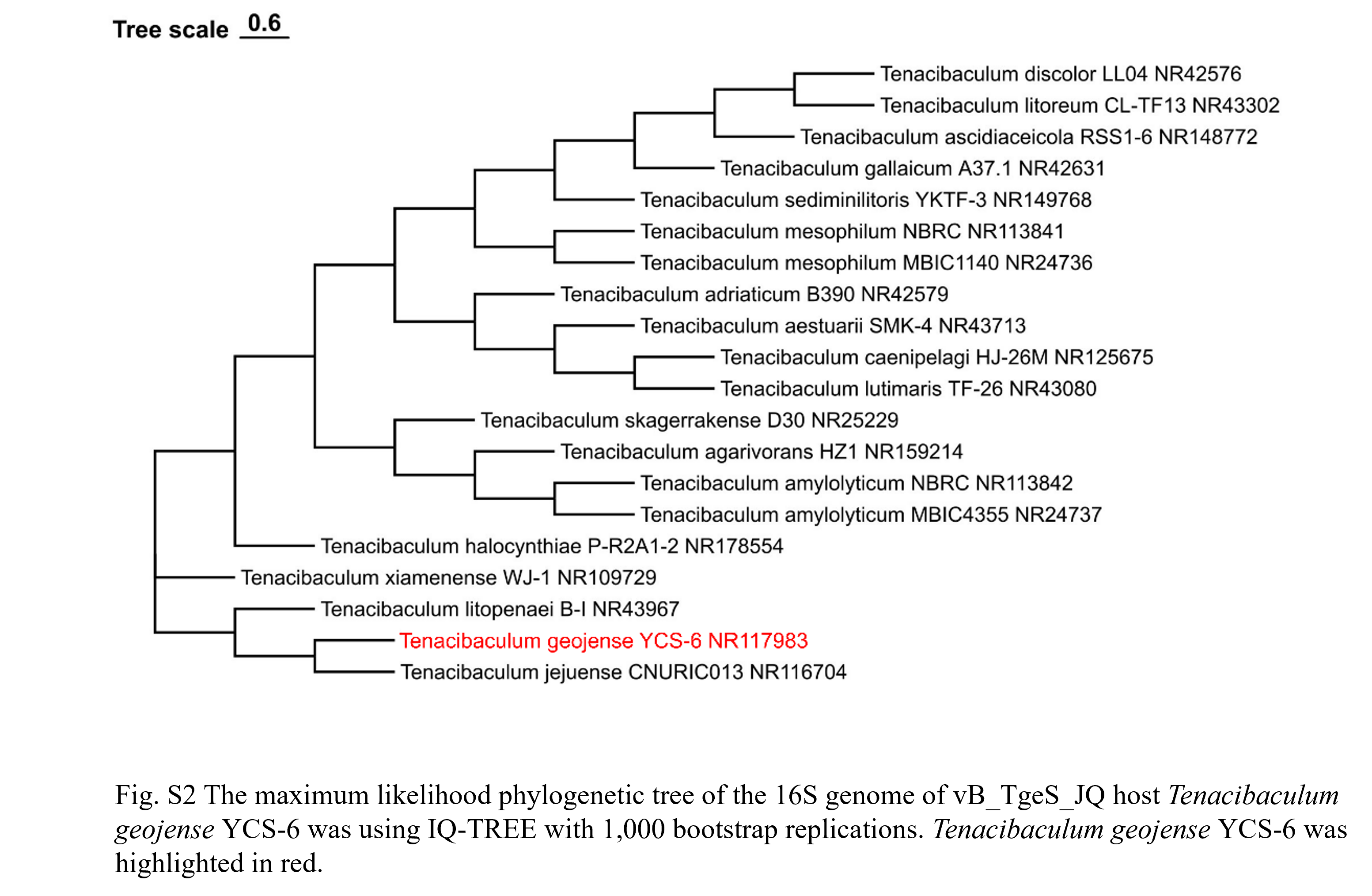

Supplement: Fig. S2 — Phylogenetic tree of host. [file aem.00367-24-s0006.tif]
